# Chemical composition, antioxidant, and enzyme inhibition activities of *Crithmum maritimum* essential oils: the first chemo-biological study for species grown in North Africa

**DOI:** 10.1038/s41598-024-74544-9

**Published:** 2024-10-25

**Authors:** Ahmed Ismail, Fatma A. El-Shibani, Hamdoon A. Mohammed, Belal O. Al-Najjar, Amany M. Korkor, Abdulnaser Kh. Abdulkarim, Rana Said, Suliman A. Almahmoud, Ghassan M. Sulaiman

**Affiliations:** 1 Pharmacognosy Department, Faculty of Pharmacy, Fayoum University, Fayoum, 63514 Egypt; 2 Department of Pharmacognosy, Faculty of Pharmacy, Benghazi University, Benghazi, Libya; 3 Department of Pharmacognosy, Faculty of Pharmacy, Assalam International University, Benghazi, Libya; 4 Department of Medicinal Chemistry and Pharmacognosy, College of Pharmacy, Qassim University, 51452 Buraydah, Qassim Saudi Arabia; 5 Department of Pharmaceutical Sciences, Faculty of Pharmacy, Al-Ahliyya Amman University, Amman, 19328 Jordan; 6 Pharmacological and Diagnostic Research Laboratory, Al-Ahliyya Amman University, Amman, 19328 Jordan; 7 Pharmacognosy Department, Faculty of Pharmacy (Girls), AL-Azhar University, Cairo, Egypt; 8 Department of Basic Medical Science, Faculty of Pharmacy, University of Tripoli, Tripoli, Libya; 9 Division of Biotechnology, Department of Applied Sciences, University of Technology, Baghdad, 10066 Iraq

**Keywords:** *Crithmum maritimum*, See fennel, Halophytes, Antioxidants, Anti-acetylcholinesterase, Anti-tyrosinase, GC-MS, Docking analysis, Chemical biology, Computational biology and bioinformatics, Molecular biology, Plant sciences

## Abstract

*Crithmum maritimum* (sea fennel), is a halophytic plant species found globally in coastal environments. This study is the first investigation into the chemical composition and biological activities of *C. maritimum* growing wildly in Jebel Akhdar, Libya. Gas Chromatography-Mass Spectrometry (GC-MS) analysis was utilized to identify and profile the plant’s volatile components; it resulted in the identification of twenty-four components, representing 99.17% of the total peaks in the GC-MS chromatogram. The analysis revealed that thymyl methyl ether, γ-terpinene, and ledene oxide, were the major volatile constituents of the plant at relative percentage levels of 56.86, 16.17, and 4.32%, respectively. The analysis also indicated substantial variations in the volatile composition of *C. maritimum* Libyan species compared to those from various geographic regions. The plant’s volatile oil quality was evaluated by investigating its in vitro antioxidant activity and the oil’s ability to inhibit acetylcholinesterase (AChE) and tyrosinase enzymes. The oil markedly scavenged the free radicals and reduced the ferric ions in the DPPH and FRAP assays at levels of 34.30 ± 0.10 and 38.90 ± 0.51 Trolox equivalents, respectively. The plant’s volatile oil has substantially reduced the AChE at the IC_50_ value of 34.43 ± 0.25 compared to its effect against tyrosinase (IC_50_ 12.449 ± 0.68). The in silico approach was used to highlight the mechanisms underlying the enzyme inhibitory effect of the plant volatile oil. The stigmastene and γ-santonin demonstrate stronger binding affinity towards AChE and tyrosinase compared to the co-crystalized controls, donepezil and tropolone. The study provides significant information for the environmental changes effect on the volatile constituents of *C. maritimum* and highlights the plant’s importance within the scope of its antioxidant and enzyme inhibition activities.

**Supplementary Information:**

The online version contains supplementary material available at 10.1038/s41598-024-74544-9.

## Introduction

The drought associated with high salinity and marsh environments is widely distributed worldwide and is expected to increase more in the coming years as a result of climate change^1^. The drought and high salinity environment are considered unfavorable conditions for plant growth and reproduction, as these conditions of water stress and high salinity induce osmotic stress, metal toxicity, and oxidative stress^2^. Overall, drought and high salinity environments impose significant physiological and biochemical challenges on plants, hindering their growth and reproductive processes^3^. These unfavorable conditions disrupt water availability, nutrient uptake, ion balance, and cellular functions, leading to reduced plant fitness and reproductive success^4,5^. There is a group of plants named halophytes that have the ability to survive in the harsh conditions of salinity and dryness^6,7^. These plants, halophytes, occupy their internal defense system against the oxidative stress developed in response to salt and dryness stresses. Higher productions of antioxidant polyphenols have been reported as part of the defensive pathways that halophytes produce to manage oxidative stress. In addition, halophytes have the ability to synthesize other secondary metabolites, e.g., volatile oils, alkaloids, saponins, and bitter principles, which seems to play a role in the halophytes management of salt stress and oxidative stress^1,8–11^. Therefore, high interest has been expressed in the halophytes as renewable sources for medicine and food.

*Crithmum maritimum* L., an Apiaceae family member, is one of the widely distributed and studied culinary halophytes. The plant is also named sea fennel and rock samphire^12^. The plant, *C. maritimum* is wildly grow beside coastlines and sand in African coast in the North and West areas. The plant also grows in Western Asia, the Mediterranean and Black Sea, the Azores, Madeira, the Canarias Islands, and Europe^13–15^.

This plant, *C. maritimum*, is used for several culinary purposes. Indeed, its leaves are abundant in several bioactive materials, including vitamins like vitamin C, bioflavonoids, and carotenoids, which have been reported for several therapeutic uses^16,17^. In addition, sea fennel is considered a low-cost raw material with high nutritional content and functional qualities that has been the subject of numerous studies to produce natural, bioactive, and health-promoting food ingredients. For instance, *C. maritimum’s* newly picked leaves and small branches are preserved in vinegar and used as condiments and appetizers. According to the portion chosen, *C. maritimum* has different medicinal uses. The aerial part’s infusion was applied to treat prostatic inflammation and nephrites. The leaf infusion has been reported to treat colic and be used for cleansing the liver. In addition, the leaves have antiscorbutic, tonic, carminative, diuretic, depurative, and vermifuge properties in traditional medicine^14,16,18,19^. Numerous studies on the use of *C. maritimum* essential oils in traditional medicine have been published^14,15,20^. Beside the carminative and anti-inflammatory applications of the plant oil, it has been widely used in cosmetic preparations^15^. The major reported volatile oil constituents of the plant have been identified as γ-terpinene, thymyl methyl ether, dillapiole^14^, α- and β-pinene, cymene, limonene^20^, sabinene, thymyl methyl oxide^15^, and β-phellandrene^21^. However, environmental conditions have induced significant quantified and qualified variations in the *C. maritimum* volatile oils^13,14,20–22^.

**Fig. 1:**
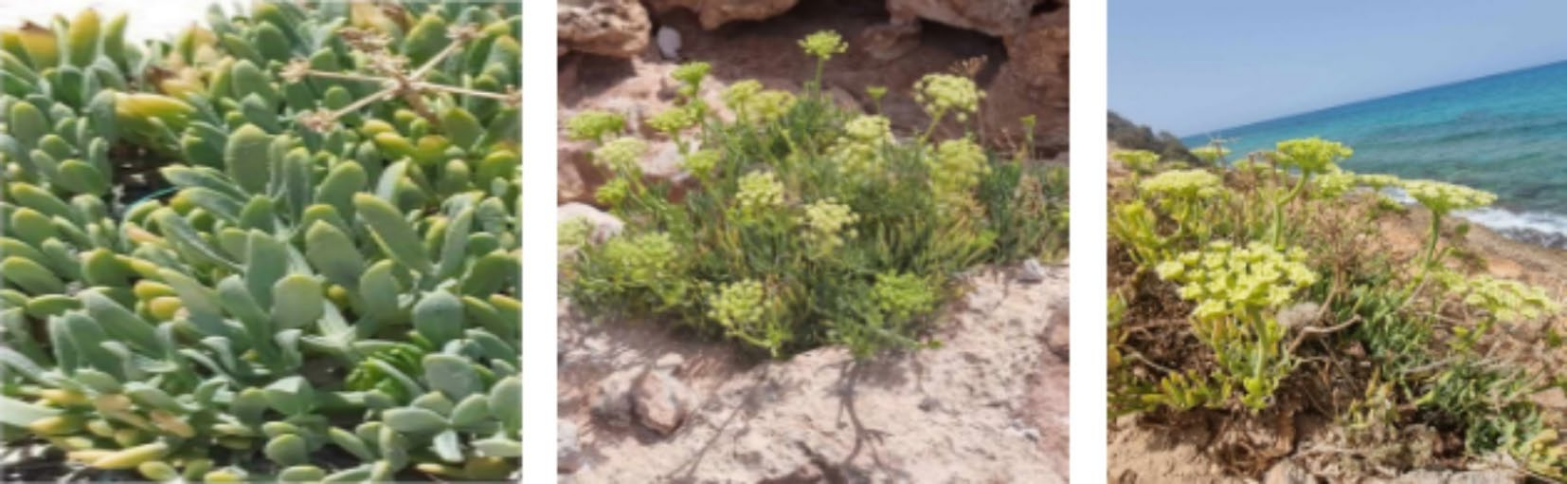
Photographs of aerial part of *C. maritimum* from the coastal region of Jebel Akhdar in east of Libya.

The present work emphasizes for the first time the chemical composition of *C. maritimum* growing in the coastal region of Jebel Akhdar in the east of Libya (Fig. 1). The plant volatile constituents were profiled using the GC-MS analysis, and the chemo-profiling findings were compared to the reported volatile constituents of the plant species growing in different locations. The quality of the plant’s volatile oils was also assessed through the evaluation of their antioxidant and enzyme inhibition activities. The study also includes in silico-based studies of the volatile constituents’ receptor binding affinity to ward AChE and tyrosinase enzymes compared to the co-crystalized controls, donepezil and tropolone.

## Materials and methods

### Plant materials

In late March of the spring, fresh aerial parts of *C. maritimum* were obtained from the eastern region of Libya. However, the national and international guidelines for the collection of wild plants, including responsible collecting, have been taken into consecration during plant collection (https://portals.iucn.org/library/efiles/documents/PP-003-En.pdf). The plant material was authenticated by Dr. Houssein Eltaguri, Department of Botany at the Faculty of Science, University of Benghazi. A sample of the material was then placed in the departmental herbarium (#DB-18890). After being air-dried for fifteen days at room temperature, the plant was preserved for future research in a tightly sealed, dark container.

### Preparation of essential oil from *C. Maritimum*

Fresh plant samples of aerial portions of *Crithmum maritimum* L. were subjected to hydro-distillation at the Pharmacognosy Department, faculty of Pharmacy, Fayoum University. One kilogram of the harvested plant material was put through a five-hour distillation process using the Clevenger equipment. The initial dry weight of the plant material was used to determine the oil output. Following drying with anhydrous sodium sulfate, the resulting oil was stored in a refrigerator until further analysis.

### Gas chromatography-mass spectroscopy study (GC-MS)

GC analysis was performed by the Nawah Scientific, Egypt, on the volatile oil of the plant, *C. maritimum*, utilizing an Agilent GC-MS device (6890 model). The fused-silica column (30 m, 0.25 mm, film thickness 0.25 μm), utilized for the oil constituent separations, was filled with cross-linked phenyl polysiloxane (HP-5MS, USA, Hewlett Packard) as the stationary phase material. At an average rate of 8 °C per minute, the temperature increased from 80 °C to 260 °C. For fifteen minutes, the final temperature was maintained. The quadrupole was at 150 °C, the ion source was at 230 °C, the ionization energy was 70 eV, and the carrier gas flow rate was kept at 0.1 mL/min. At 3.62/scan, the scan range covered 40–500 m/z. By comparing the mass spectral fragmentation pattern of the constituents with the NIST database (John Wiley Library, # 229119), the constituents were also identified.

### Antioxidant assays

The antioxidant assays were performed at the National Research center in Egypt. The analysis was performed in triplicate and calculated as mean ± SD.

#### DPPH (2,2-diphenyl-1-picrylhydrazyl) scavenging activity

According to literature^23^ 150 µL of freshly prepared DPPH reagent (prepared by dissolving 2 mg with 51 ml of methanol HPLC grade) was mixed with 5 µL of the plant volatile oil, which was prepared at the concentration of 10 mg/mL. The mixture was kept for 30 min in dark. At 517 nm, the change in DPPH color was assessed in three independent measurements, and the DPPH’s equivalent to Trolox was determined. Data are represented as mean ± SD.

#### Ferric reducing antioxidant power (FRAP) assay

According to published method^24^, evaluation of antioxidant potential was processed. The TPTZ working reagent (190 µL) was added to 10 µL of the volatile oil (10 mg/mL) on a 96-well plate. The mixture was then allowed to sit at room temperature for 30 min before being measured at 593 nm. The reagent was made up of acetate buffer (300 mM PH = 3.6), TPTZ (10 mM in 40 mM HCl), and FeCl_3_ (20 mM). Trolox equivalent in milligrams was used to measure the FRAP extract activity.

#### Oxygen radical absorbance capacity (ORAC) assay

The assay was performed as shown in the published method^25^. 30 µL of fluoresceine (100 nM) was incubated with 10 µL of the volatile oil for 10 min at 37 °C. Three cycles of fluorescence measurement (485 EX, 520 EM, nm) with a cycle length of 90 s were carried out for the background measurement. Subsequently, 70 µL of newly produced 2,20-Azobis(2-amidinopropane) dihydrochloride (AAPH) (300 mM) was added to each plate. For 60 min, the fluorescence measurements (485 EX and 520 EM nm) were maintained (40 cycles, each lasting 90 s).

### Enzymes inhibitors assays

The enzymatic assays were performed at the National Research center in Egypt. The analysis was performed in triplicate and calculated as mean ± SD.

#### Determination of tyrosinase inhibitory properties

The determination of tyrosinase inhibitory activity was investigated with the aid of a spectrophotometer. The tyrosinase inhibitory activity of the sample was compared with arbutin as a reference substance^26^.

A mixture of 2 mL L-tyrosine solution (0.244 mM) and 0.9 mL 50% methanol solution of inhibitor was made in aqueous phosphate buffer (pH 6.8; I 0.01 M), an equivalent volume of 50% methanol solution was used for the control sample in place of the inhibitor solution. By adding 0.1 mL of the aqueous mushroom tyrosinase solution (0.1 mg/mL), L-tyrosine was oxidized. For ten minutes, the oil sample and control combination were incubated at 37 °C. Using spectrophotometry, the dopachrome appearance was tracked at 475 nm. IC_50_ was used to measure the impact on tyrosinase inhibition. The following formula was utilized to calculate the percentage of tyrosinase activity inhibition:

$$\%{\text{ inhibition = 100 - \{ (A475 of test sample / A475 of control) * 100\} }}$$

The absorbance values in the absence and presence of inhibitors were A sample 475 and A control 475.

#### Determination of ACE inhibitory effect

Ellman method was utilized for Acetylcholinesterase Inhibitor Screening^27^, wherein 5,5-dithiobis(2-nitrobenzoic acid) (DTNB) and thiocholine generated by acetylcholinesterase combine to form a yellow color. The product color intensity, which is determined at 412 nm, is a direct function of the sample’s enzyme activity. Transfer 45 µL of AChE to the 96-well plate’s duplicate wells. Add 1 µL of AChE. Once the test chemicals are dissolved, add 5 µL of the solvent. Add 5 µL of the test compounds to the remaining wells. After 15 min, incubate. For every response effectively: Combine 0.5 µL of Chromogen, 1 µL of Substrate, and 154 µL of assay Buffer. To each well, add 150 µL of this working reagent (step 6). Incubate the plate at room temperature; after 0, 10 and 30 min. Review color change by reading the absorbance at 412 nm. The inhibition of the AChE was calculated according to the reported method^27^.

### Docking analysis

In order to inspect the binding affinity of the 25 identified compounds (Table 1), molecular docking simulations were performed against tyrosinase (PDB ID: 2Y9X) and acetylcholinesterase (AChE) (PDB ID: 4EY7)^28–30^. AutoDock 4.2.6 was utilized for this part of the study according to the methods described earlier by our group with slight modifications^31,32^. Briefly, all protein structures were prepared using BIOVIA Discovery Studio 16.1 by removing water molecules and complexed co-structures. Complexed inhibitors (donepezil and tropolone, for acetylcholinesterase (AChE) and tyrosinase, respectively) were separated from the crystal structures to be used as control ligands. Using AutoDockTools 1.5.6, Kollman charges and polar hydrogen atoms were assigned to the proteins. On the other hand, the 3D conformers of the compounds were downloaded from NCBI PubChem database (pubchem.ncbi.nlm.nih.gov) and Gasteiger charges were assigned accordingly. A grid box with the size of 15^3^ Å was set with the coordinates of -13.988, -43.906, and 27.108 as *x*, *y*,*z*, respectively, for the AChE, and with the same size at -10.043, -28.28, and − 43.443 as *x*, *y*,*z*, respectively, for the tyrosinase. Simulations were carried out using 100 Lamarckian Genetic Algorithm runs with the default parameters. Conformations with the lowest free energy of binding (LEB) and the most populated cluster were selected for further analysis. Interactions’ analyses were carried out using BIOVIA Discovery Studio 16.1.

### Statistical analysis

The data and measures obtained were analyzed and presented as mean ± standard deviation for at least three tests.

## Results and discussion

### Essential oil profiling

The GC-MS analysis of the essential oil of *C. maritimum* was performed in the current study to profile the volatile constituents for the plant species growing in the coastal region of east Libya and demonstrate the qualitative and quantitative variations in these constituents compared to the constituents of the plant species growing in different regions and under different climatic conditions. The plants’ responses to environmental conditions and biotic stresses, including their liability to biosynthesis-specific constituents, have been documented in several reports^33–35^. There is also a literature proving that volatile constituents of plants have great sensitivity, compared to other plants secondary metabolites, to the abiotic and biotic stresses that affect the plants during their growth^25,36–38^. In the current findings, twenty-five compounds were identified in the volatile oil sample of the plant (Table 1). Among the identified compounds, the most abundant compound in *C. maritimum* as profiled by the GC-MS was the thymyl methyl ether with a relative percentage of 56.86%, indicating its dominance in the plant volatile composition (Table 1). This compound has also been reported as one of the major volatile constituents of *C. maritimum* species growing in different regions^14,22,39,40^. However, the Libyan species of the plant has the highest amount of the compound. For instance, the relative concentration of thymyl methyl ether in the *C. maritimum* Tunisian species was found at 20.13–40.40% ^14,22^; in the Turkish growing plant, the percentages of the compound were found at 7.7–29.8% ^40,41^; in the species from Portugal, the percentages of the compound were 12.90–15% ^42^; and in Palagonia, the percentages of the compound were found at 25.48% ^39^. The variations in the thymyl methyl ether and other volatile concentrations of the plant, *C. maritimum*, growing in different locations, indicated the plant’s sensitivity to changes in the environmental conditions related to its growing areas which is expecting a variation in its biological activity comparing with the same species in other geographical locations.

Furthermore, γ-terpinene (16.17%) was also found at a high relative percentage in the current Libyan species. The compound has also been identified as a major constituent in several *C. maritimum* species growing around the world; however, its percentages in those species were flocculated up and down^15,22,39^. Compared to the relative percentages of γ-terpinene in plant species around the world, current findings indicate a relatively lower concentration in the Libyan species of *C. maritimum*. For instance, γ-terpinene has been found at percentages of 33.60%, 19.3–30.62%, and 8.8–32.4% in the species growing in Portugal, Tunisia, and Turkey, respectively^15,22,40^.

Elucidating the GC-MS analysis also revealed the presence of ledene oxide, γ-guaiene and terpinen-4-ol at the concentration levels of 4.32, 3.32 and 2.89, respectively, and at relatively higher concentrations compared to other identified constituents, except for the existence of γ-terpinene and thymyl methyl ether. The analysis also revealed the presence of several other biologically active volatile constituents in considerable percentages. For example, carvacrol and thymol, the antioxidant and antimicrobial phenolic monoterpene volatile oils^25,43^, were found at the relative percentages levels of 0.91, and 1.15%, respectively. All the previously mentioned compounds and the other identified volatile constituents of the plant in Table 1 such as germacrene D (2.17%) and cuparene (0.69%) are contributing to the overall complexity of the plant aroma.

**Fig. 2:**
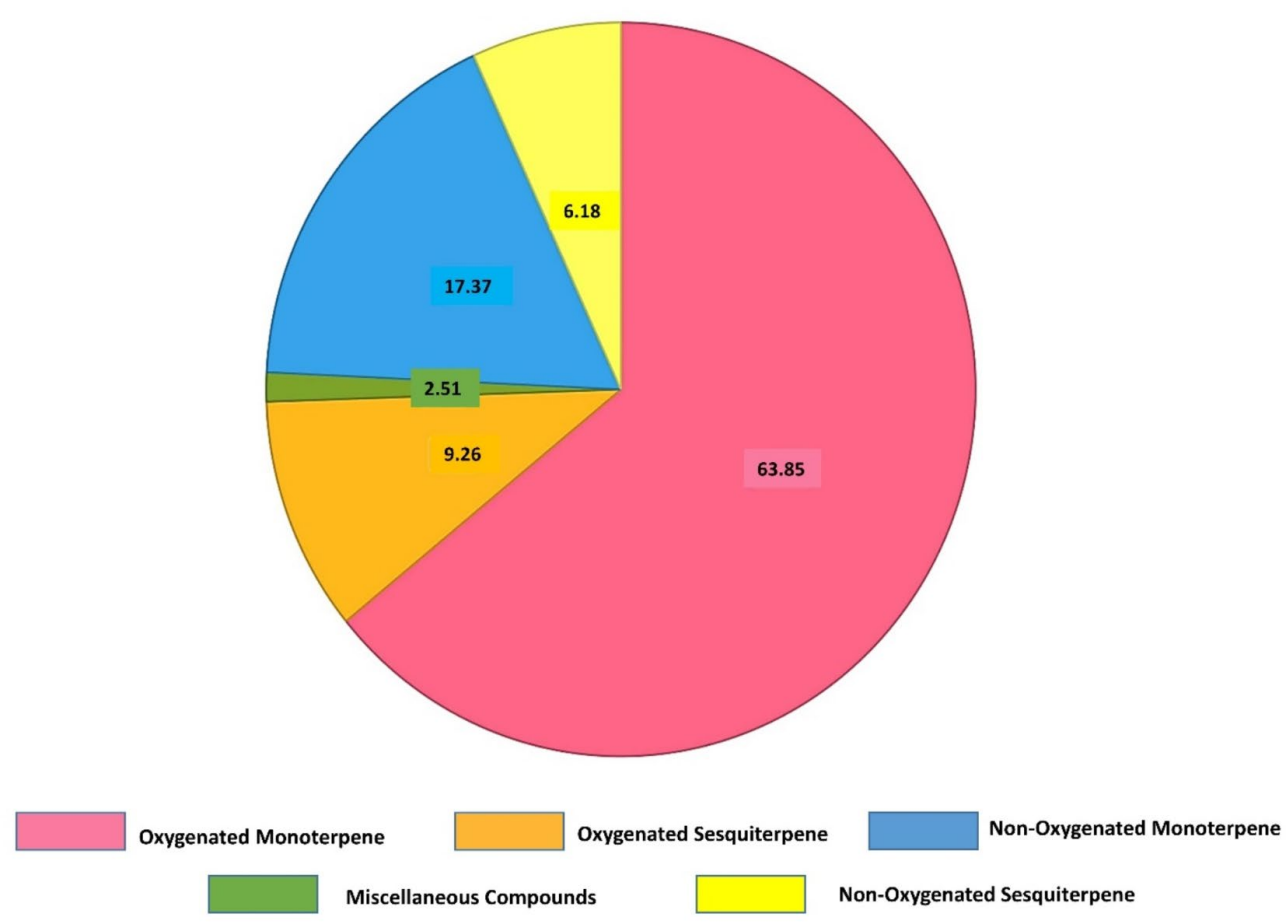
Relative percentages of the collective classes of volatile oils in *Crithimum maritimum*.

Grouping of the identified compounds indicated the presence of the oxygenated monoterpenes in significant combined higher concentration (63.85%) compared to other groups of constituents including the non-oxygenated monoterpenes, the oxygenated sesquiterpenes, and the non-oxygenated sesquiterpenes, which were calculated with the relative percentages of 17.37, 9.26, and 6.18%, respectively (Fig. 2).

The presence of these bioactive volatile constituents in the plant implicated its importance in both the food and medicine application. These bioactive volatile constituents could be participated in the beneficial application of the plant in the cosmetic preparation and in traditional medicine including its application as carminative and anti-inflammatory agent^14,15,20^. GC-MS Chromatogram of the essential oil of *C. maritimum* in **supplementary file**.

**Table 1:** Volatile oil constituents of *Crithimum maritimum* growing in the coastal area of Libya.

No	KI	Compound	M. formula	Percentage
1.	1031	β- Phellandrene	C_10_H_16_	1.2
2.	1040	γ-terpinene	C_10_H_16_	16.17
3.	1162	Terpinen-4-ol	C_10_H_18_O	2.89
4.	1205	Thymyl methyl ether	C_11_H_16_O	56.86
5.	1248	Phellandral	C_10_H_16_O	1.03
6.	1255	Carvone	C_10_H_14_O	1.01
7.	1289	Carvacrol	C_10_H_14_O	0.91
8.	1290	Thymol	C_10_H_14_O	1.15
9.	1480	Germacrene D	C_15_H_24_	2.17
10.	1502	Cuparene	C_15_H_22_	0.69
11.	1577	Isospathulenol	C_15_H_24_O	0.36
12.	1593	Salvial-4(14)-en-1-one	C_15_H_24_O	0.54
13.	1608	Neoclovenoxid-alcohol	C_15_H_24_O	1.58
14.	1619	Spathulenol	C_15_H_24_O	0.77
15.	1621	Apiole	C_12_H_14_O_4_	0.46
16.	1634	Ledene oxide-(II)	C_15_H_24_O	4.32
17.	1695	Ent-Germacra-4(15),5,10(14)-trien-1β-ol	C_15_H_24_O	0.93
18.	1763	Aristolene epoxide	C_15_H_24_O	0.23
19.	1764	γ-Guaiene	C_15_H_24_	3.32
20.	2053	2,4,6,8,10,12-Hexamethyl-1,12-tridecadiene	C_19_H_36_	0.86
21.	2131	Hexahydrofarnesyl acetone	C_18_H_36_O	0.32
22.	2202	α- Santonin	C_15_H_18_O_3_	0.53
23.	3030	Stigmastene	C_29_H_50_	0.42
24.	3184	Oleic acid	C_19_H_36_O_2_	0.45
**Total**				**99.17**
Oxygenated monoterpene				63.85
Oxygenated sesquiterpenes				9.26
Non-oxygenated monoterpene				17.37
Non-oxygenated sesquiterpenes				6.18
Miscellaneous compounds				2.51

### Antioxidant activity of *C. Maritimum*

**Fig. 3:**
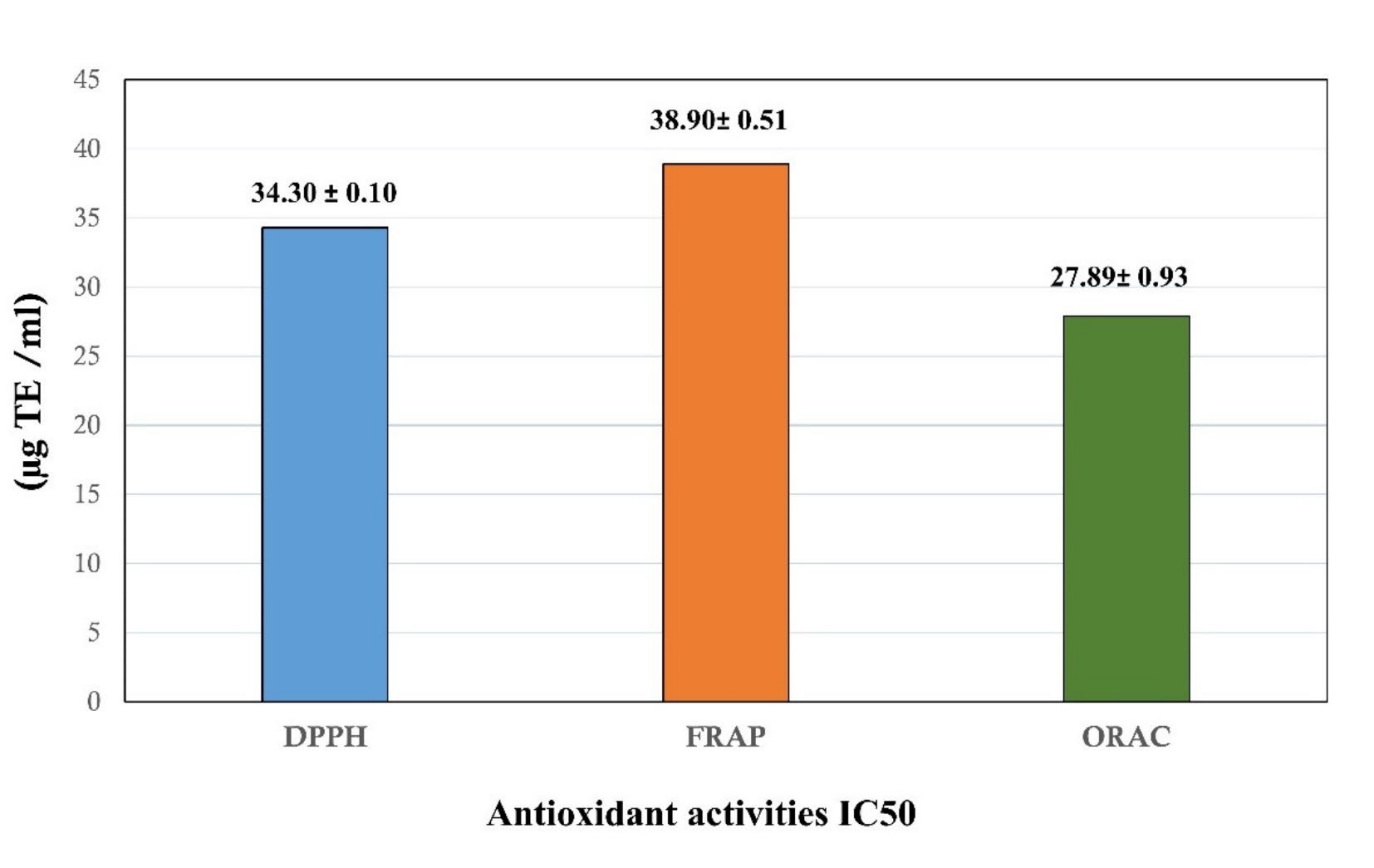
Antioxidant activities of essential oils from *C. maritimum. The values indicated are three equivalent measurements’ means ± SD. TE: Trolox equivalent; FRAP: Ferric reducing antioxidant power; ORAC: Cupric reducing antioxidant capacity; DPPH: 2*,*2-diphenyl-1-picrylhydrazyl*.

As part of the *C. maritimum* quality evaluation, the antioxidant activity of the volatile oils of the plant has been measured using three different in vitro assays, i.e., DPPH, FRAP, and ORAC. These methods were selected to evaluate the transition metals reducing power and free radical capturing effect of the plant volatile oils^23^. The results demonstrated in Fig. 3 indicated that the plant volatile oils have exerted substantial DPPH and peroxyl radical scavenging effect in the DPPH and ORAC assays at the values of 34.30 ± 0.10 and 27.89 ± 0.93 µM TE/ mg of the plant volatile oils, respectively. *C. maritimum* volatile oils also exhibited remarkable reducing power effect to the ferric ions in the FRAP test at the level of 38.90 ± 0.51 µM TE/ mg of the plant volatile oils.

The results also indicated the higher antioxidant activity of the *C. maritimum* Libyan species compared to the plant species growing in different locations. For instance, the extract of the plant species growing in Croatia has demonstrated lower reducing power (FRAP) and peroxyl radical scavenging effects compared to the Libyan species^18^. This antioxidant potential may related to variable contents of the oil and its enrichment of thymyl methyl ether and other volatile concentrations as γ-terpinene comparing with the same species in other geographical locations. The plant species growing in Tunisia have also exerted antioxidant effects against DPPH free radicals and reduced power for ferric ions at the values of IC_50_ 0.44–3.3 and EC_50_ 2.44–3.08, respectively^22^, which seem to have lower effects compared to the present antioxidant results recorded for the Libyan species of the plant. However, an exact comparison to the reported antioxidant results is difficult due to the variations in the assay analysis, as the reported methods calculated the IC_50_/EC_50_^22^, and in the current methods, the antioxidant results were expressed as Trolox equivalents (Fig. 3).

### Tyrosinase and AChE inhibitory effect of *C. Maritimum* essential oil

Enzyme inhibition is an important therapeutic approach for the treatment of a number of issues. For example, AChE has been linked to neurological disorders that are implicated in the etiology of Alzheimer’s disease (AD) and is involved in the hydrolysis of neurotransmitters, specifically acetylcholine, which terminates neurotransmission. On the other hand, melasma, age spots, freckles, and other skin hyperpigmentation problems are thought to be best treated by regulating the manufacture of melanin. Using common in vitro assays, the anti-tyrosinase and anti-AChE properties of *C. maritimum* essential oil were assessed; the results of Enzyme inhibitory activity of essential oils from *C. maritimum* are shown in Table 2.

**Table 2:** Enzyme inhibitory activity of essential oils from *C. Maritimum*.

Sample	Tyrosinase (mg Ar/ml)	AChE (mg GALAE/ml)
**IC**_**50**_	12.449 ± 0.68	34.43 ± 0.25

*Values expressed are means ± S.D. of three equivalent measurements. AChE: Acetylcholinesterase; Ar: Arbutin equivalent.*

Acetylcholinesterase (AChE) inhibitors find extensive biological use in the treatment of neurological conditions, particularly Alzheimer’s disease. By impeding AChE activity, these inhibitors elevate acetylcholine levels in the brain, temporarily improving cognitive function in affected individuals. This approach aims to address the neurotransmitter imbalance characteristic of Alzheimer’s, offering symptomatic relief^44,45^.

The mechanism of action of acetylcholinesterase (AChE) inhibitors in Alzheimer’s disease treatment revolves around the enhancement of cholinergic neurotransmission. In Alzheimer’s, there is a deficiency of the neurotransmitter acetylcholine due to increased breakdown by AChE. AChE inhibitors, such as donepezil, rivastigmine, and galantamine, work by blocking the activity of AChE^46^. By inhibiting AChE, these drugs allow acetylcholine to accumulate in the synaptic cleft, facilitating increased stimulation of cholinergic receptors. This elevated acetylcholine level helps improve neurotransmission and temporarily alleviates cognitive symptoms associated with Alzheimer’s disease, such as memory loss and cognitive decline. While AChE inhibitors do not halt the progression of Alzheimer’s, they provide symptomatic relief and can enhance cognitive function, thereby improving the quality of life for individuals affected by the disease.

Tyrosinase enzyme inhibitors, on the other hand, are commonly explored in skincare and cosmetics due to their role in melanin synthesis. By impeding tyrosinase activity, these inhibitors can mitigate hyperpigmentation and even out skin tone. The efficacy of such inhibitors depends on factors like formulation, concentration, and individual skin characteristics^47^.

The mechanism of action of tyrosinase inhibitors in skin disease treatment lies in their ability to regulate melanin production. Tyrosinase is a key enzyme involved in the melanin synthesis pathway. Melanin is the pigment responsible for skin, hair, and eye color. Overactivity of tyrosinase can lead to hyperpigmentation disorders, such as melasma, age spots, and certain types of hyperpigmentation^48^. Tyrosinase inhibitors, commonly used in skincare products, work by interfering with the enzymatic activity of tyrosinase. By inhibiting tyrosinase, these compounds reduce the production of melanin, leading to a decrease in pigmentation and a more even skin tone. This is particularly beneficial in treating conditions where excessive melanin production results in uneven skin pigmentation^49^. The application of tyrosinase inhibitors in skincare underscores their significance in addressing cosmetic concerns related to hyperpigmentation and promoting a more uniform complexion.

Both AChE and tyrosinase enzyme inhibitors underscore the importance of enzyme modulation in diverse fields, ranging from neuroscience to dermatology, showcasing their potential therapeutic applications.

It was previously reported that EOs from different Croatian sea fennel were highly efficient against cholinesterase enzymes, the flower extract from sea fennel proved to have strong vasodilatory properties. Anti-acetylcholinesterase activities of essential oils of aerial parts of Tunisian *Crithmum maritimum* L. showed nearly the same activity 31.16 ± 0.012 mg/ml compared with the species under investigation; 34.43 ± 0.25 mg/ml^19^. A previous study evaluated the Anti-Tyrosinase activity of essential oils of *Crithmum maritimum* L. from France and Croatia, it reported the inactivity of the French one while (IC50 = 649 µg/mL) of Croatian sample. In the other side Libyan species activity is 12.449 ± 0.68, which indicates that geographical variability played an essential role in oil composition and its activity^50^. In vitro and intracellular antioxidant capacity of thymyl methyl ether was evaluated in Druce leaves and it exhibited a significant intracellular antioxidant capacity and considered as hepatoprotective agent^51^. These characteristics may indicate the possibility of using sea fennel in the culinary, medicinal, and other industries. Particularly, the flowers and stems have not always been used.

One of the most prominent *Artemisia campestris* essential oil constituents; γ-terpinene was reported both tyrosinase (38.36 ± 3.86%) and AChE inhibition (53.95 ± 5.55%) compared to Kojic acid and Galantamine, respectively^52^.

Molecular docking study on apiole from essential oil of *Petroselinum crispum* (Mill, ) Fuss in study of some Moroccan Apiaceae species, it showed an AChE inhibition with binding energy (-5.9 kcal/mol) for the interactions with the acetylcholinesterase^53^.

The biological features under investigation were found to be affected differently by the essential oils isolated from aerial parts of Libyan sea fennel, which contain wide variety of phytochemicals of different classes where thymyl methyl ether, γ-terpinene and apiole were the most abundant ones.

### Docking results

**Fig. 4:**
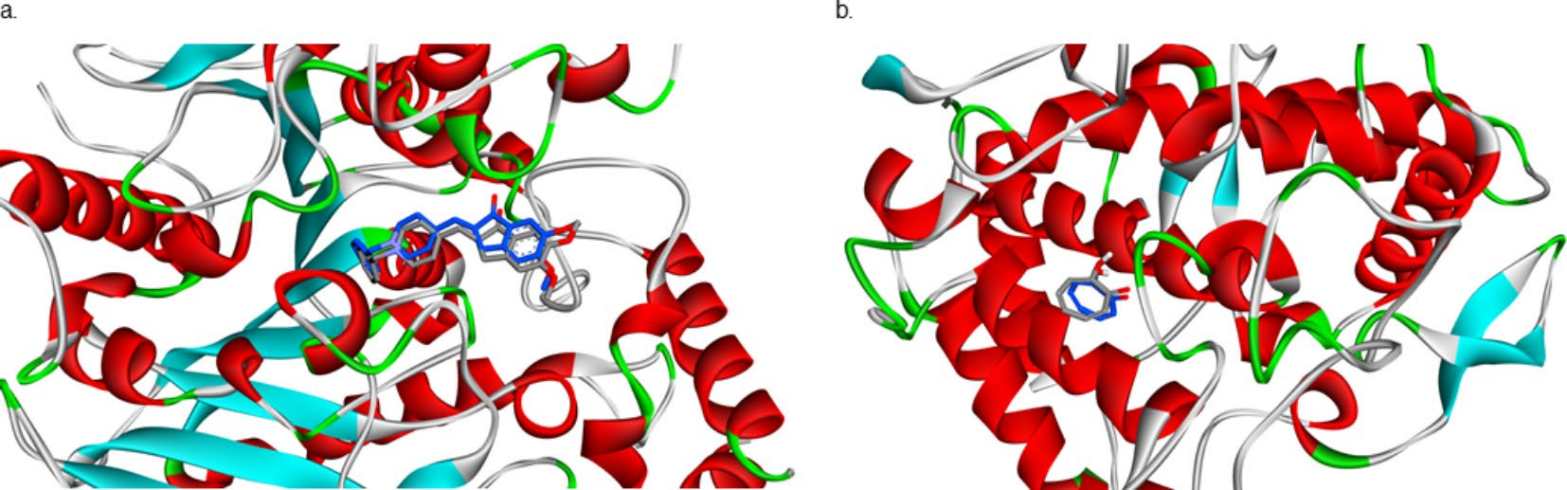
Stick representations of co-crystal structures in grey color and the docked configurations in blue color against (**a**) Acetylcholinesterase and (**b**) Tyrosinase enzymes. Generated by BIOVIA Discovery Studio visualizer.

This study investigated the potential of *Crithmum maritimum* essential oils as inhibitors for two important therapeutic targets: the tyrosinase and acetylcholinesterase. To validate the docking procedure, the two co-crystal structures of Donepezil and tropolone were redocked, yielding an RMSD value of 0.69 and 1.96 Å, respectively, as represented in Fig. 4. It is proposed that an RMSD values, falling beneath the 2 Å limit, validates the reliability of the docking protocol for subsequent examinations^54^. The results suggest that several compounds exhibit promising binding affinities towards these targets, with binding energies ranging from − 5.81 to -13.14 Kcal/mol (Supplementary file).

**Fig. 5:**
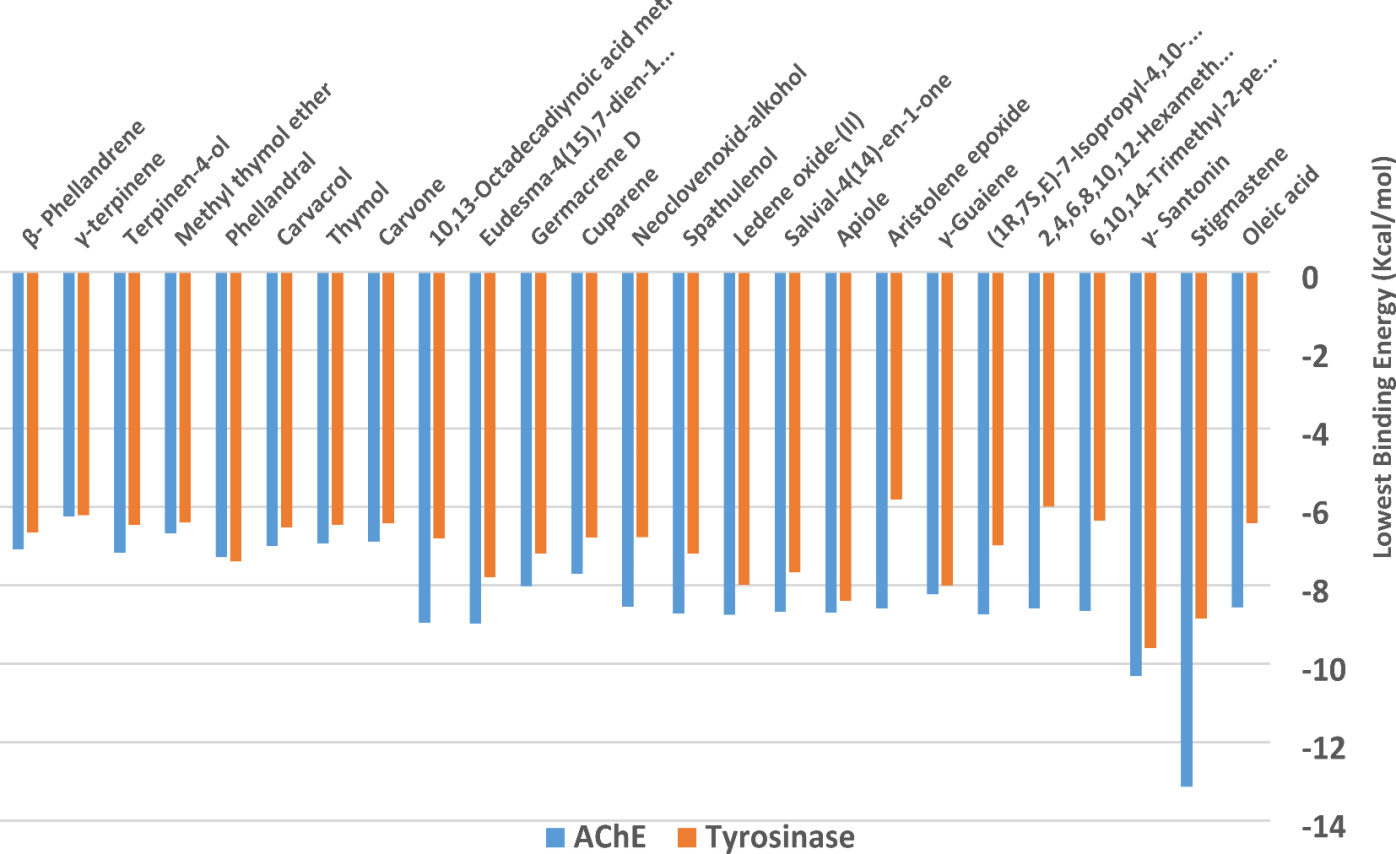
Column chart showing the lowest binding energy for the *Crithmum maritimum* essential oils against tyrosinase and acetylcholinesterase (AChE).

Column chart summarizes the molecular docking results of the compounds against both tyrosinase and acetylcholinesterase (AChE) provide significant insights into their potential inhibitory activities. The binding energies for tyrosinase range from − 6.24 to -13.14 kcal/mol. Compounds such as γ-santonin, stigmastene, and Apiol exhibit remarkably low binding energies, suggesting strong binding to tyrosinase. On the other hand, the binding energies for AChE range from − 5.81 to -9.6 kcal/mol. Interestingly, again both stigmastene and γ-santonin demonstrate the strongest binding to AChE among the compounds tested (Fig. 5).

**Fig. 6:**
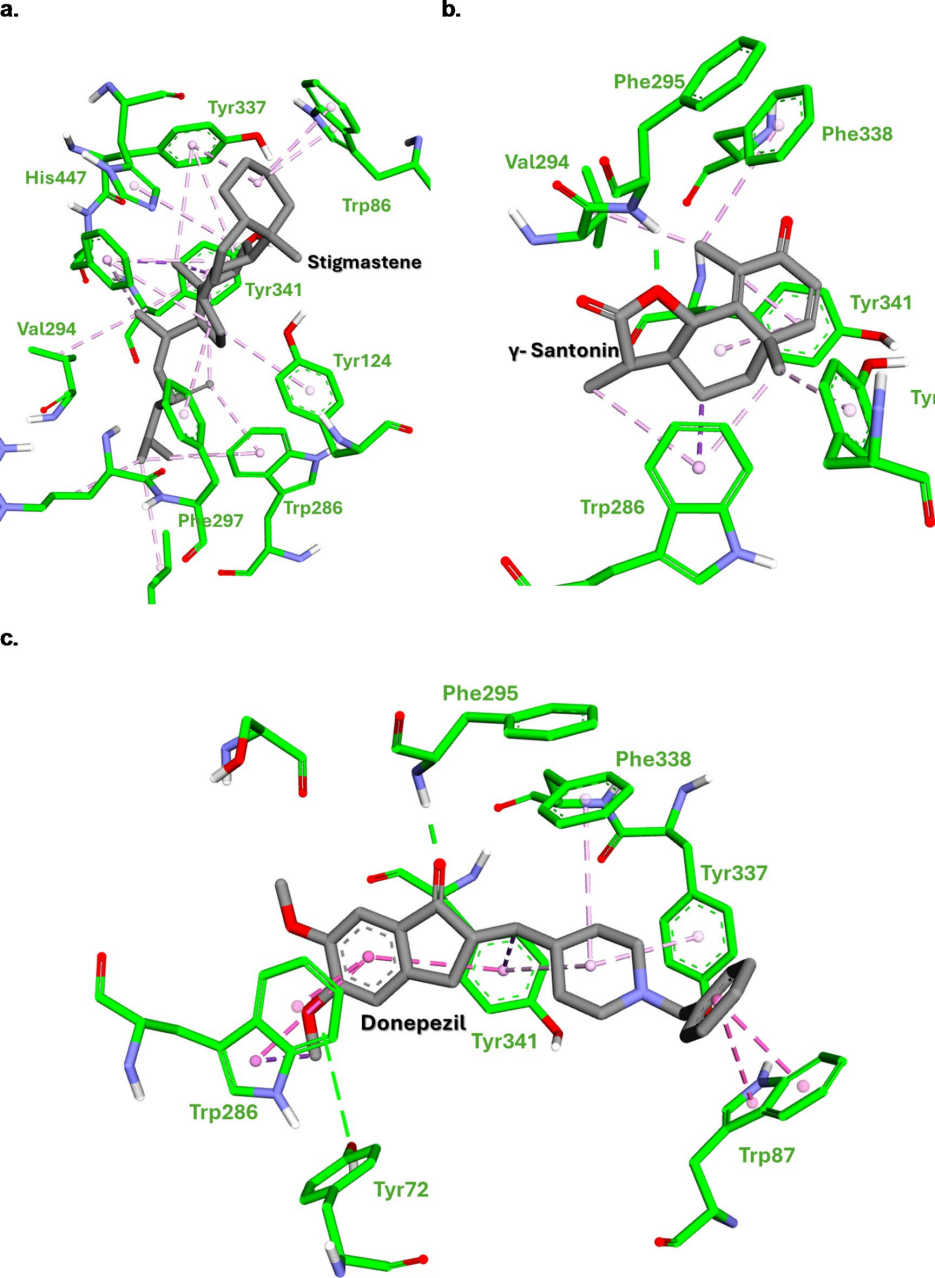
Stick representation of (**a**) stigmastene, (**b**) γ- santonin, and (**c**) donepezil, docked within acetylcholinesterase (PDB ID: 4EY7) binding site. Generated by BIOVIA Discovery Studio visualizer.

**Fig. 7:**
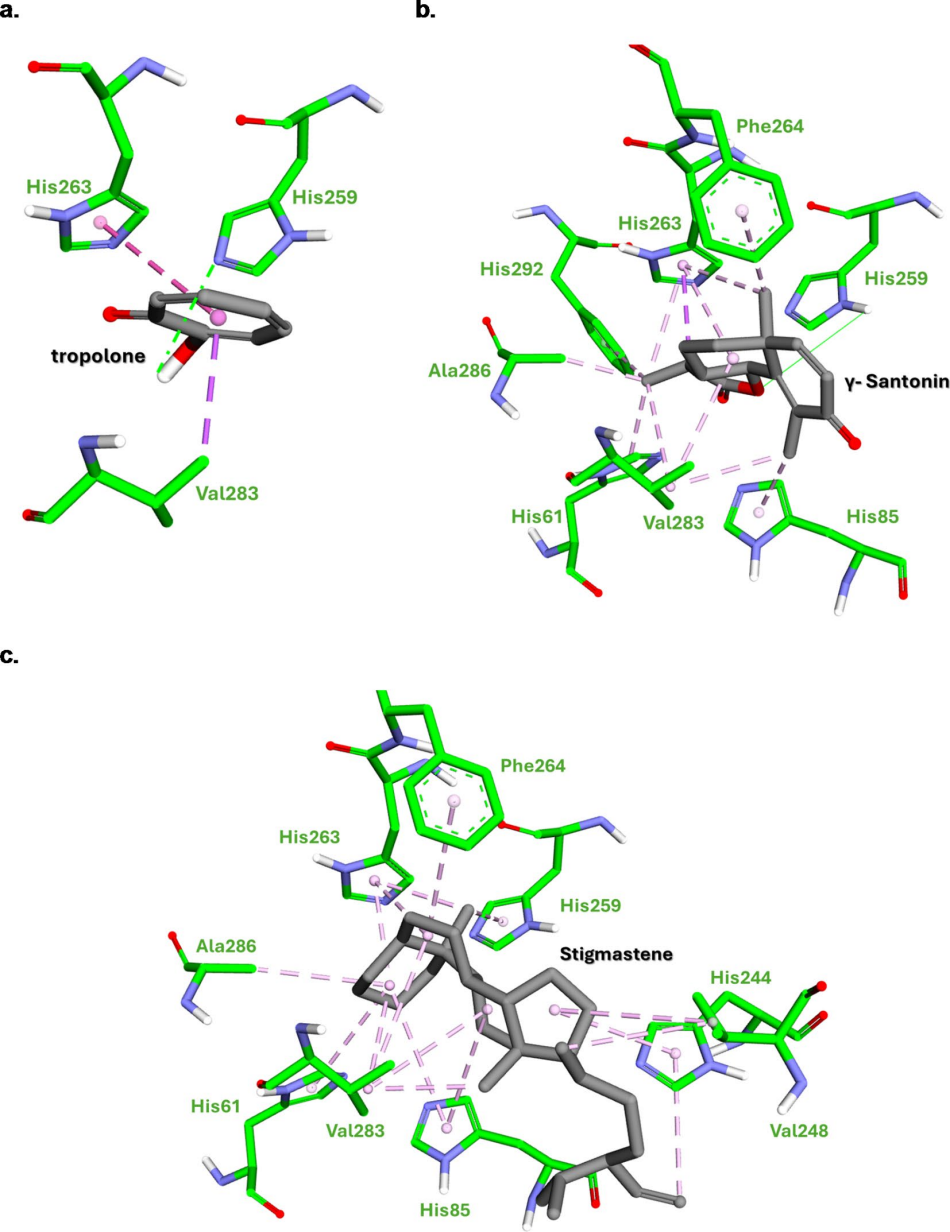
Stick representation of (**a**) tropolone, (**b**) γ- santonin, and (**c**) stigmastene, docked within tyrosinase (PDB ID: 2Y9X) binding site. Generated by BIOVIA Discovery Studio visualizer.

According to the initial docking results, γ-santonin and stigmastene emerged as frontrunners due to their exceptional binding affinities towards both enzymes. A more detailed investigation into the intermolecular interactions within the binding pocket of these compounds could elucidate the specific molecular forces governing their remarkable binding behaviors. Table 3; Figs. 6 and 7 showed the detailed intermolecular interactions for both compounds in the respected binding sites.

**Table 3:** Docking scores (LBE, Kcal/mol) and putative interacting amino acids in the binding pocket of compounds exhibiting highest affinity for acetylcholinesterase and tyrosinase enzymes.

Compound	Acetylcholinesterase				Tyrosinase			
	LBE (Kcal/mol)	Interacting amino acids			LBE (Kcal/mol)	Interacting amino acids		
		H-Bond	Hydrophobic	π - π		H-Bond	Hydrophobic	π - π
Stigmastene	-13.14	None	Trp86, Tyr124, Trp286, Val294, Phe297, Tyr337 Tyr341, His447	None	-8.85	None	His61, His85, His244 Val248, His259, His263, Phe264, Val283 Ala286	None
γ- Santonin	-10.31	Phe295	Tyr124, Trp286, Val294, Phe338	Tyr341	-9.6	His259	His85, His61, His263, Phe264, Val283, Ala286, His292	None
Donepezil	-11.04	Tyr72, Phe295	Tyr337 Phe338 Tyr341	Trp87 (Aromatic), Trp286 (Aromatic), Tyr341(Aromatic),	-	-	-	-
Tropolone	-	-	-	-	-4.53	His259	Val283, His263	None

Stigmastene displayed the highest binding affinity among the three (-13.14 kcal/mol) against acetylcholinesterase enzyme, followed by donepezil (-11.04 kcal/mol) and γ-santonin (-10.31 kcal/mol). Interestingly, stigmastene lacks predicted hydrogen bonds with AChE, while both donepezil and γ-santonin form hydrogen bonds with key amino acid residues. This suggests that stigmastene’s exceptional binding might be driven by other factors like hydrophobic interactions. Additionally, both stigmastene and γ-santonin interact with a similar set of hydrophobic amino acids (Trp86, Tyr124, Trp286, Val294, Phe297, Tyr337, Tyr341, His447). Donepezil also interacts with some of these residues but lacks interactions with Val294 and His447. The extensive hydrophobic interactions observed for stigmastene could be a significant contributor to its strong binding affinity. Even though none of the compounds were predicted to form π-π interactions with AChE residues. Donepezil, however, performs aromatic interactions with Trp87, Trp286, and Tyr341, which might contribute to its binding. These docking results highlight the importance of both hydrophobic interactions and hydrogen bonding in AChE inhibition.

On the other hand, stigmastene and γ-santonin demonstrate stronger binding affinity towards tyrosinase compared to the co-crystalized control, tropolone. While γ-santonin forms a hydrogen bond with His259, similar to tropolone, its overall stronger binding could be attributed to additional hydrophobic interactions with other amino acid residues. Again, stigmastene, despite lacking hydrogen bonds, exhibits the strongest binding due to its extensive hydrophobic interactions across the active site. Both stigmastene and γ-Santonin engage in extensive hydrophobic interactions with tyrosinase residues, indicating favorable binding.

These findings suggest the potential of stigmastene and γ-santonin as tyrosinase and acetylcholinesterase inhibitors,

## Conclusion

The present work evaluated the phytochemical contents and quality of the *Crithmum maritimum* volatile oil as new findings for the plant species growing in different location, i.e., Jabal Akhdar in Libya. The results demonstrated in this study indicated chemical variations between the *C. maritimum* species growing in Libya and those species growing elsewhere in the world, appeared in the higher concentration of thymyl methyl ether in the Libyan species compared to the species growing in different location. The plant’s volatile oil has exerted potential qualities which were evaluated by investigating its in vitro antioxidant activity and the oil’s ability to inhibit acetylcholinesterase (AChE) and tyrosinase enzymes. Specific volatile constituents, i.e., stigmastene and γ-santonin demonstrate high binding affinity towards AChE and tyrosinase compared to the co-crystalized controls, donepezil and tropolone. The study emphasizes the significance of *C. maritimum* in terms of its antioxidant and enzyme inhibitory capabilities and offers important information regarding the impact of environmental changes on the volatile constituents of the plant.

## Electronic supplementary material

Below is the link to the electronic supplementary material.

Supplementary Material 1

## Data Availability

Data is provided within the manuscript or supplementary information files.
