# Supplementary material for: Chemical composition, antioxidant, and enzyme inhibition activities of Crithmum maritimum essential oils: the first chemo-biological study for species grown in North Africa
Source: Sci Rep. 2024 Oct 25;14:25318. doi: 10.1038/s41598-024-74544-9 (PMC11512020; doi:10.1038/s41598-024-74544-9)
Supplement: Supplementary file 1 — Supplementary Material 1 [file 41598_2024_74544_MOESM1_ESM.docx]

**Supplementary file**


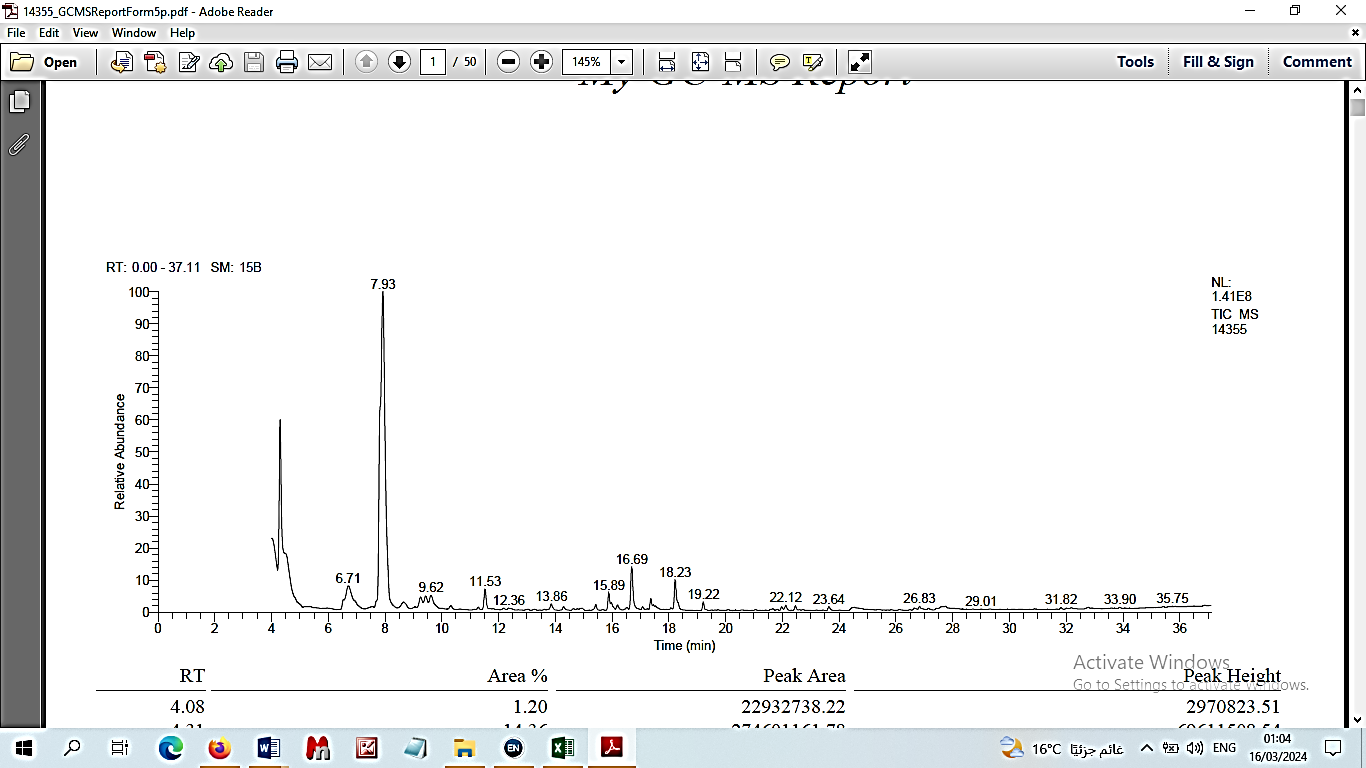


**Figure 1. GC-MS Chromatogram of the essential oil of *C. maritimum***

**Figure. 2 Standard curve of Acetylcholinesterase Inhibitor Screening**


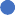

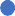

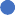

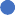

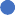

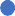


300000

250000

y = 262.02x + 10600

R² = 0.9928

200000

150000

100000

50000

0

0

200

400

600

Trolox concentration (µM)

800

1000

1200

#

RFU

#

#

**Figure. 3 Standard curve of ORAC antioxidant activity assay.** (RFU; relative fluorescence units)

**Figure. 4 Standard curve of DPPH antioxidant activity assay**

**Figure. 5 Standard curve of FRAP antioxidant activity assay**

**Table 1. Lowest Binding Energy scores (Kcal/mol) for the *Crithmum maritimum* essential oils against tyrosinase and acetylcholinesterase (AchE).**

|  | **Compound** | **LBE (Kcal/mol)** | |
| --- | --- | --- | --- |
|  |  | **Tyrosinase** | **AchE** |
| 1 | β- Phellandrene | -7.08 | -6.65 |
| 2 | γ-terpinene | -6.24 | -6.21 |
| 3 | Terpinen-4-ol | -7.17 | -6.46 |
| 4 | Methyl thymol ether | -6.67 | -6.4 |
| 5 | Phellandral | -7.28 | -7.39 |
| 6 | Carvacrol | -6.99 | -6.52 |
| 7 | Thymol | -6.93 | -6.45 |
| 8 | Carvone | -6.88 | -6.42 |
| 9 | 10,13-Octadecadiynoic acid methyl ester | -8.96 | -6.8 |
| 10 | Eudesma-4(15),7-dien-1á -ol | -8.97 | -7.79 |
| 11 | Germacrene D | -8.02 | -7.19 |
| 12 | Cuparene | -7.7 | -6.78 |
| 13 | Neoclovenoxid-alkohol | -8.54 | -6.77 |
| 14 | Spathulenol | -8.72 | -7.19 |
| 15 | Ledene oxide-(II) | -8.75 | -7.98 |
| 16 | Salvial-4(14)-en-1-one | -8.68 | -7.67 |
| 17 | Apiole | -8.7 | -8.4 |
| 18 | Aristolene epoxide | -8.59 | -5.81 |
| 19 | γ-Guaiene | -8.23 | -8 |
| 20 | (1R,7S,E)-7-Isopropyl-4,10-dimethylenecyclodec-5-enol | -8.74 | -6.98 |
| 21 | 2,4,6,8,10,12-Hexamethyl-1,12-tridecadiene | -8.59 | -5.99 |
| 22 | 6,10,14-Trimethyl-2-pentadecanone | -8.65 | -6.35 |
| 23 | γ- Santonin | -10.31 | -9.6 |
| 24 | Stigmastene | -13.14 | -8.85 |
| 25 | Elaol | -9.36 | -7.59 |
| 26 | Oleic acid | -8.56 | -6.41 |
| 27 | Donepezil (Docking control for AChE) | -11.04 | - |
| 28 | Tropolone (Docking control for Tyrosinase) | - | -4.53 |
